# Less-Intelligent and Unaware? Accuracy and Dunning–Kruger Effects for Self-Estimates of Different Aspects of Intelligence

**DOI:** 10.3390/jintelligence10010010

**Published:** 2022-02-05

**Authors:** Gabriela Hofer, Valentina Mraulak, Sandra Grinschgl, Aljoscha C. Neubauer

**Affiliations:** Institute of Psychology, University of Graz, Universitätsplatz 2, 8010 Graz, Austria; valentina.mraulak@gmail.com (V.M.); sandra.grinschgl@uni-graz.at (S.G.); aljoscha.neubauer@uni-graz.at (A.C.N.)

**Keywords:** self-knowledge, accuracy, self-estimates, Dunning–Kruger effect, overestimation, intelligence, cognitive abilities, metacognition

## Abstract

People’s perceptions of their intelligence correlate only moderately with objective intelligence measures. On average, people overestimate themselves. According to the popular Dunning–Kruger effect, this is particularly true for low performers: across many domains, those in the lowest quartile overestimate their abilities the most. However, recent work using improved statistical approaches found little support for a Dunning–Kruger effect in general intelligence. We investigated accuracy and Dunning–Kruger effects for self-estimates of general, verbal, numerical, and spatial intelligence—domains that differed in how well they can be judged in the past. A total of 281 participants completed self-estimates and intelligence measures online. Self-estimates showed mostly moderate correlational accuracy that was slightly higher for numerical intelligence and lower for verbal intelligence. Across domains, participants rated their intelligence as above average. However, as their intelligence was indeed high, this was not an overestimation. While standard analyses indicated Dunning–Kruger effects in general, verbal, and spatial intelligence, improved statistical methods only yielded some support for one in verbal intelligence: people with lower verbal intelligence tended to have less self-knowledge about it. The generalizability of these findings is limited to young, highly educated populations. Nevertheless, our results contribute to a growing literature questioning the generality of the Dunning–Kruger effect.

## 1. Introduction

Do people have an accurate sense of how intelligent they are? Research suggests that this might not necessarily be the case (for an overview, see Neubauer and Hofer 2020). Self-estimates of intelligence and other abilities often correlate only moderately with corresponding objective performance criteria (Freund and Kasten 2012; Zell and Krizan 2014). Looking at the direction of this miscalibration, many studies suggest that people tend to overestimate themselves (e.g., Gignac and Zajenkowski 2019). A striking example for this tendency is the often-reported above-average or better-than-average effect, according to which people, on average, believe their abilities to be above average (Alicke and Govorun 2005). Other research indicates that the tendency towards overestimation depends on the underlying ability level with less capable people showing the highest degree of overestimation—a finding known as the Dunning–Kruger effect (Kruger and Dunning 1999).

There are many good reasons to be interested in the accuracy of self-estimates of abilities in general and of intelligence in particular: self-estimates are often-used in career counselling (Holling and Preckel 2005) and can affect career decisions beyond that (see also Freund and Kasten 2012). Basing one’s life decisions on incorrect self-estimates could have adverse consequences (see also Ackerman and Wolman 2007): people who have chosen a job based on an overestimation of their abilities could face constant overload, while those who underestimate themselves might not take career opportunities due to unwarranted fears of failure. Some authors have also reported that more accurate self-views are related to higher psychological adjustment (Kim et al. 2010; Kim and Chiu 2011), although others have found overestimation (Dufner et al. 2018; He and Côté 2019) or just generally positive self-views (Humberg et al. 2019) to be more beneficial. These associations of self-views and their accuracy/positivity with outcomes as important as decision-making or psychological adjustment make this an interesting field of study. For these reasons, we aimed to take a closer look at the different effects related to the accuracy of self-estimates—that is, correlational accuracy, above-average effects, the direction of misestimation, and Dunning–Kruger effects. More specifically, we were interested in the accuracy of self-estimates of general intelligence and three intelligence facets that are included in most common theories of intelligence (e.g., Cattell 1963; Jäger 1984; Thurstone 1938): verbal, numerical, and spatial intelligence.

### 1.1. Correlational Accuracy of Self-Estimates of Intelligence

Considering common effect-size guidelines (Cohen 1992), the correlation between self-estimated and measured intelligence—sometimes termed “relative accuracy” (Schraw 2009)—is moderate: a meta-analysis across 41 studies estimated it to be *r* = .33 (Freund and Kasten 2012), a number that is similar to what was found across meta-analyses on the accuracy of self-estimates of various abilities (Zell and Krizan 2014). This is surprisingly low if one considers self-estimates to tap into the same latent construct as performance tests. Some authors even concluded that self-estimates of abilities might have more in common with personality traits than with the underlying ability (Herreen and Zajac 2018; Neubauer and Hofer 2021). However, research also showed that correlational accuracy differs between intelligence facets: in their meta-analysis, Freund and Kasten found self-estimates of numerical but not spatial or verbal intelligence to be more accurate than those of general intelligence. In a similar vein, recent studies reported a correlational accuracy of between *r* = .4 and *r* = .5 for numerical intelligence and smaller—in some cases even negligible—correlations for spatial and particularly verbal intelligence (Neubauer et al. 2018; Neubauer and Hofer 2021). The reasons for these differences between domains do not seem to be clear at the moment. Moreover, some have argued that focusing on correlations alone when studying accuracy is far from optimal, as the insights to be gained from them are limited (Dunning and Helzer 2014). As an example, correlational accuracy alone says nothing about the direction of potential miscalibration (or “bias”; (Schraw 2009) of self-estimates.

### 1.2. Above-Average Effects and the Miscalibration of Self-Estimates of Intelligence

According to a large body of research, people likely have a general tendency to be overly confident of their abilities. To state some examples, people, on average, rate their abilities in a variety of domains including sense of humor (Horrey et al. 2015), and also driving skills (Kruger and Dunning 1999), to be above average (for a discussion of above-average effects, see Alicke and Govorun 2005). These effects are also present for intellectual abilities: based on two large and representative data sets, Heck et al. (2018) reported that 65% of Americans think that their general intelligence is above average. In another study, college students rated themselves to be above the average student on all of Gardner’s (1999) multiple intelligences—including linguistic, logical-mathematical, and spatial intelligence (Visser et al. 2008). When comparing self-estimated to measured intelligence, one study found that people overestimated themselves by as much as 30 IQ-points (Gignac and Zajenkowski 2019). However, if everybody was to overestimate themselves to a similar degree, this should still result in high correlational accuracy as self-estimates would keep their rank-order validity (i.e., highly intelligent people would still rate themselves as more intelligent than less-intelligent people; see also (Vazire 2010). Thus, there are likely individual differences in the strength and direction of miscalibration, raising the question of potential moderators: what characterizes people who underestimate themselves, overestimate themselves, or have an accurate view of their own abilities?

### 1.3. Dunning–Kruger Effects

The probably most discussed moderator of the miscalibration of self-estimates of abilities is the person’s underlying ability level in the domain in question (see also Gignac and Zajenkowski 2020; for discussions of other moderators, see Freund and Kasten 2012; Zell and Krizan 2014). Kruger and Dunning (1999) proposed that the individuals with the lowest abilities in a domain are also the ones showing the strongest tendency towards overestimation (i.e., the Dunning–Kruger effect). According to the authors, low performers lack the metacognitive competence to correctly assess their shortcomings: “Not only do they reach mistaken conclusions and make regrettable errors, but their incompetence robs them of the ability to realize it” (Kruger and Dunning 1999, p. 1132). The effect has received a lot of attention, both from the scientific community and the general public: Dunning–Kruger effects were reported in many studies across a variety of domains (for a summary, see Dunning 2011), including intelligence (von Stumm 2014). In popular media, the Dunning–Kruger effect has been widely discussed in connection with topics such as the Trump presidency (e.g., Devega 2020; Pressler 2017) or conspiracy theories related to the COVID-19 pandemic and climate change (e.g., Shepherd 2020).

Notably, research on the Dunning–Kruger effect has also faced quite some criticism for its data-analytical methods. In their seminal study, Kruger and Dunning (1999) first split their sample into quartiles based on participants’ objective performance and then compared the average self-estimated and measured performance within each quartile (for comparable approaches, see, e.g., Ehrlinger et al. 2008; von Stumm 2014; West and Eaton 2019). The authors’ results indicated that people in the lowest quartile overestimated their performance greatly, while those in the top quartile underestimated themselves slightly. Several authors have proposed that these results could also be due to statistical artifacts (e.g., Krajč and Ortmann 2008; Nuhfer et al. 2016). Specifically, some have proposed that result patterns indicative of a Dunning–Kruger effect could be driven by a combination of the above-average effect and regression to the mean (Ackerman et al. 2002; Krueger and Mueller 2002). Based on regression to the mean (e.g., Campbell and Kenny 1999), in imperfectly correlated variables—such as self-estimated and measured intelligence—extreme values on one variable (e.g., measured intelligence) are often accompanied by values that are closer to the mean on the other variable (e.g., self-estimated intelligence). In the case of self-estimates of abilities, this mean is also elevated (above-average effect). Thus, not only will low performers overestimate and high performers underestimate themselves (regression to the mean), but the degree of miscalibration will also be higher for low performers (Krueger and Mueller 2002). Simulation studies showed that regression to the mean alone (Ackerman et al. 2002) or in conjunction with an above-average effect (Gignac and Zajenkowski 2020) could indeed produce results that many would consider supportive of Dunning–Kruger effects.

Gignac and Zajenkowski (2020) recently proposed that future studies on the Dunning–Kruger effect should apply analyses that do not rely on artificial categorization of continuous data and that are not confounded by regression to the mean and the above-average effect. The authors have suggested that at least two types of analyses fulfill these criteria: in the first approach, one tests the residuals from a linear regression where self-estimates are predicted from performance for heteroscedasticity. If participants on the lower end of the ability spectrum were indeed to show higher miscalibration, their absolute residuals should also be higher than those of participants on the higher end of the ability spectrum. As a second approach, the authors proposed to look at nonlinear effects: for a Dunning–Kruger effect, the association between measured and self-estimated ability should be higher the more capable people are, that is, there should be a positive quadratic effect. Gignac and Zajenkowski (2020) also collected data on self-estimated and measured general intelligence to compare these statistical approaches to the classical quartile-based approach. While their data were indeed indicative of a Dunning–Kruger effect when analyzed with the classical approach, the effect of measured intelligence on self-estimated intelligence was essentially linear with no significant heteroscedasticity of residuals. This raises the question of how robust Dunning–Kruger effects truly are. Moreover, despite the often-reported differences in correlational accuracy between intelligence facets, we are not aware of any study that distinguished between facets when investigating Dunning–Kruger effects. As both Dunning (2011) and Gignac and Zajenkowski (2020) proposed that some domains might be more susceptible to Dunning–Kruger effects than others, we think that such a comparison between intelligence facets could provide interesting insights.

### 1.4. The Present Study

In the present, preregistered, study, we investigated the accuracy of self-estimates of general, verbal, numerical, and spatial intelligence. Following other authors’ recommendations (e.g., Dunning and Helzer 2014; Schraw 2009), we considered different operationalizations of accuracy. Specifically, we investigated correlational accuracy, above-average effects, the direction of miscalibration, and Dunning–Kruger effects. We anticipated positive correlations between self-estimates and respective objective measures, with a medium relationship for general intelligence (e.g., Freund and Kasten 2012), a medium-to-large relationship for numerical intelligence, and a small relationship for verbal and spatial intelligence (e.g., Neubauer et al. 2018; Neubauer and Hofer 2021). We also predicted above-average effects, that is, that, on average, people would self-estimate all aspects of their intelligence to be above 100 IQ-points (e.g., Heck et al. 2018; Visser et al. 2008). We further expected that people would overestimate themselves on all intelligence measures (e.g., Gignac and Zajenkowski 2019). Additionally, we wanted to know whether Dunning–Kruger effects can be found for the different aspects of intelligence when using (1) the conventional statistical methods applied in this line of research (e.g., Kruger and Dunning 1999; West and Eaton 2019), and (2) the statistical methods suggested by Gignac and Zajenkowski (2020). We had no specific expectations for this research question. In a final (exploratory) research question, we wanted to analyze how people who are more intelligent in one area than another derive their self-estimate of general intelligence: do they think of their strengths (i.e., the area in which they have the highest IQ) or rather their weaknesses when estimating their overall IQ? Such a focus on one’s strengths would be in line with findings that people base their self-judgments in a given trait on their own, self-serving definitions of said trait (Dunning and Cohen 1992; Dunning and McElwee 1995).

## 2. Materials and Methods

We follow current standards (Simmons et al. 2012) in reporting how we determined our sample size, all data exclusions, and all measures in the study. The preregistration (https://doi.org/10.17605/OSF.IO/HMJSW) as well as code, data, and an appendix containing additional analyses (https://doi.org/10.17605/OSF.IO/MJD8E) are available via the OSF.

### 2.1. Participants

Based on sample-size recommendations by Gignac and Zajenkowski (2020), we aimed to test at least 200 participants, but we also decided to collect data for as long as time constraints would permit. This resulted in a total of 298 participants. Nine participants were excluded because they used unauthorized resources (e.g., a calculator) during the intelligence tests, and another six because they used an incorrect response format for the test of numerical intelligence. We excluded two additional participants because their self-estimates (possible range 55 to 145 IQ-points) were outside of the possible range of the intelligence tests (verbal: 59.5–131.5 IQ-points; numerical: 68.5–131.5 IQ-points; spatial 65.5–140.5 IQ-points), even though they solved all or none of the items correctly.^1^ Thus, the final sample consisted of 281 participants (67.3% female, 31.3% male, 1.4% diverse) between 18 and 40 years (*M* = 23.77, *SD* = 4.96). The sample—consisting mainly of university students (85.1%; 45.2% psychology students)—was recruited via the university mailing list and social media. The level of education within our sample was high: 68.3% had a high-school degree and another 27.8% already had a college/university degree. All participants were offered written ipsative feedback on their self-estimates and their performance on the intelligence tests. This entailed a general description of the different intelligence facets as well as two plots (one for self-estimated and one for measured abilities), depicting the participant’s (self-estimated/measured) individual strengths and weaknesses as compared to their mean score across all intelligence facets. Psychology students could additionally gain course credits. Participants gave their informed consent prior to participating and the study procedure had been approved by the ethics committee of our university.

### 2.2. Materials and Methods

#### 2.2.1. Intelligence

Verbal, numerical, and spatial intelligence were each assessed with a 20-item-subtest of the German intelligence test *Intelligenz-Struktur-Analyse* (ISA; (Fay et al. 2001). We used *commonalities* (time limit: 6 min) to measure verbal intelligence, *number series* (time limit: 11 min) to measure numerical intelligence, and *figure completion* (time limit: 7 min) to measure spatial intelligence. To obtain IQ scores, we converted the number of correctly solved items to *T*-scores for each intelligence domain using the original test norms for the total sample and then transformed the resulting *T*-scores. We averaged across the three specific IQs to calculate general intelligence. Reliabilities (Cronbach’s α; internal consistency) were good for general (α = .85), numerical (α = .86), and spatial (α = .78) intelligence, and lower but still acceptable for verbal intelligence (α = .65).

#### 2.2.2. Self-Estimated Intelligence

We applied two different methods to measure self-estimates of intelligence. First, participants had to estimate their own verbal, numerical, and spatial intelligence on a multi-item questionnaire (Neubauer et al. 2018; Neubauer and Hofer 2021). Here, participants responded to statements such as “Compared to others, I have a very broad vocabulary.” (verbal intelligence) on a Likert scale ranging from 1 (not true at all) to 5 (exactly true). The different items (10 items for verbal intelligence; 9 items each for numerical and spatial intelligence) referred to different aspects of the respective intelligence facet; the last item of each subscale referred to a global self-estimate in the respective domain (e.g., “I am very talented in the verbal domain.”). Internal consistencies were good to excellent (*α*_verbal_ = .83; *α*_numerical_ = .95; *α*_spatial_ = .89). We used scale means for testing our research questions. 

Another common approach to measure self-estimates of intelligence is to let participants directly estimate their IQ (e.g., Furnham 2001; von Stumm 2014), which offers the advantage of allowing for a direct comparison to measured IQ. Thus, participants also self-estimated their general IQ as well as their verbal, numerical, and spatial IQ, each on a single item ((Hofer et al. 2022;) for the items, see our OSF project). Before doing so, participants read a brief text explaining the intelligence distribution in the population. In addition, we presented this distribution on a graph including the labels “slightly impaired”, “below average”, “average”, “above average”, and “highly gifted”. Participants were then asked to rate their IQ—compared to the general population—on a slider ranging from 55 IQ-points (slightly impaired) to 145 IQ-points (highly gifted).

### 2.3. Procedure

We implemented this online-study with the survey software Unipark. Participants first read and confirmed the consent form as well as a note stating that they are not allowed to distribute any of the tests. After responding to socio-demographic questions (age, gender, education, and current employment), participants estimated their own intelligence, first with the multi-item questionnaires and then with the single items. Like Gignac and Zajenkowski (2020; however, cf. Kruger and Dunning 1999), we presented the intelligence measures—in our case the tests commonalities, number series, and figure completion—after the self-estimate questionnaires. Finally, participants answered five control questions regarding the use of unauthorized tools (dictionary, search engines, other people, calculator, or other tools) when taking the intelligence tests. A single test session took between 45 and 60 min.

## 3. Results

We conducted all our analyses in R (R Core Team 2021). As there were doubts regarding the normality of some variables (for histograms see Figures A1 and A2 in the Appendix on the OSF) and some of the distributional regression assumptions (see Figures A3–A10 in the Appendix), we reported bootstrapped confidence intervals for 2000 samples wherever possible.

### 3.1. Descriptive Statistics and Intercorrelations

Table 1 displays descriptive statistics and intercorrelations of all main variables. Objective intelligence measures, single-item IQ estimates, and multi-item intelligence self-estimates each showed mostly small-to-medium correlations among the different domains. For all three types of measures, these correlations were descriptively somewhat higher between the numerical and spatial domain than between the two and the verbal domain. Notably, single- and multi-item self-estimates within the same domain correlated at between .65 and .76 with each other.

### 3.2. Linear Associations between Self-Estimated and Measured Intelligence

We first analyzed the correlational accuracy of self-estimates of intelligence. In a secondary research question, we were further interested in potential differences in accuracy between intelligence self-estimates with social comparison (i.e., the self-estimated IQs) and without social comparison (i.e., the multi-item self-estimates). Due to the higher number of underlying items, the multi-item self-estimates benefited from higher reliability, thus allowing for higher possible correlations (e.g., Epstein 1983). For better comparability, we also report correlations for the last item of each multi-item scale, which was a global estimate of the respective intelligence facet. As shown in Table 2, self-estimates generally showed positive correlations with the respective measured intelligence facet. These correlations were significant in all cases but one (self-estimated verbal IQ). Most correlations were small to medium, with the highest correlations for numerical intelligence and the lowest correlations for verbal intelligence. Table 2 further depicts that, within the same domain, the different self-estimate measures showed similar levels of correlational accuracy. We conducted two-sided Williams’ *t*-tests (Williams 1959; see Steiger 1980) between correlations within the cocor-package (Diedenhofen and Musch 2015) to test for differences in accuracy between self-estimate measures. None of these tests reached significance (all *p* ≥ .065; for detailed statistics, see Table A2 in the Appendix A). As an additional measure of accuracy, we also computed absolute agreement between self-estimated and measured IQs (i.e., two-way mixed intraclass correlation coefficients; see Koo and Li 2016). Results were similar to those of Pearson correlations: general intelligence: ICC (280, 280) = .25, 95% CI [.15; .34], *p* < .001; verbal intelligence: ICC (280, 280) = .10, 95% CI [.00; .20], *p* = .045; numerical intelligence: ICC (280, 280) = .40, 95% CI [.31; .48], *p* < .001; spatial intelligence: ICC (280, 280) = .28, 95% CI [.19; .37], *p* < .001.

### 3.3. Above-Average Effects and Miscalibration

Comparing the self-estimated IQs from Table 1 to the population average of 100 with one-sample *t*-tests indicated above-average effects across all domains, people self-estimated their verbal (*M* = 109.15; *t*(280) = 13.61, *p* < .001, *d* = 0.81, 95% BCa CI [0.67; 0.95]), numerical (*M* = 103.35; *t*(280) = 4.59, *p* < .001, *d* = 0.27, 95% BCa CI [0.16; 0.39]), spatial (*M* = 102.9; *t*(280) = 4.60, *p* < .001, *d* = 0.27, 95% BCa CI [0.16; 0.38]), and general (*M* = 109.29; *t*(280) = 16.56, *p* < .001, *d* = 0.99, 95% BCa CI [0.84; 1.13]) IQs to be significantly above 100 points. Of note, participants’ measured IQs were also significantly above 100 across domains (verbal IQ: *M* = 11.96, *t*(280) = 17.89, *p* < .001, *d* = 1.07, 95% BCa CI [0.86; 1.26]; numerical IQ: *M* = 113.28, *t*(280) = 16.99, *p* < .001, *d* = 1.01, 95% BCa CI [0.85; 1.18]; spatial IQ: *M* = 102.11, *t*(280) = 2.44, *p* = .015, *d* = 0.15, 95% BCa CI [0.03; 0.26]; general IQ: *M* = 108.78, *t*(280) = 16.25, *p* < .001, *d* = 0.97, 95% BCa CI [0.81; 1.13]).

Next, we tested the direction of miscalibration in separate analyses for general intelligence and intelligence in the three domains. To see whether potential effects of miscalibration differed across the content domains, we analyzed them together in a 3 (domain: verbal, numerical, and spatial) × 2 (measure: IQ and self-estimated IQ) repeated-measures ANOVA with Greenhouse–Geisser correction. It resulted in two significant main effects (domain: *F*(1.96, 550.07) = 70.97, *p* < .001, η^2^_g_ = .067; measure: *F*(1, 280) = 39.48, *p* < .001, η^2^_g_ = .022) and a significant interaction (*F*(1.93, 540.61) = 50.10, *p* < .001, η^2^_g_ = .035). We probed the interaction with pairwise comparisons (Bonferroni-adjusted alpha: .05/3 = .017) to investigate the degree of over- or underestimation in the different domains. Interestingly, people underestimated their numerical intelligence (*t*(280) = −11.95, *p* < .001, *d* = −0.71, 95% BCa CI [−0.87; −0.55]). While they also showed a small underestimation effect for their verbal intelligence, it was not significant after applying the Bonferroni correction (*t*(280) = −2.10, *p* = .037, *d* = −0.13, 95% BCa CI [−0.25; −0.00049]). Finally, there was no significant miscalibration for spatial intelligence (*t*(280) = 0.87, *p* = .384, *d* = 0.05, 95% BCa CI [−0.06; 0.18]). An additional pairwise *t*-test also showed no significant miscalibration for general intelligence (*t*(280) = 0.75, *p* = .455, *d* = .04, 95% BCa CI [−0.07; 0.16]).

### 3.4. Dunning–Kruger Effects

#### 3.4.1. Conventional Statistical Approach

**Figure 1:**
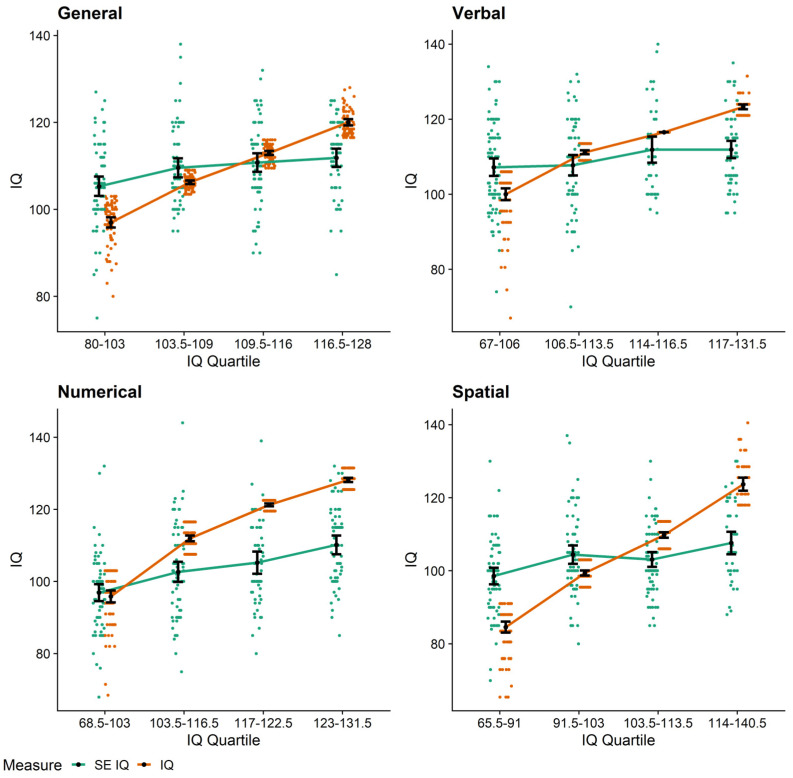
Dunning–Kruger effects: mean self-estimated (green) and measured (orange) intelligence for intelligence quartiles. Colorful dots indicate jittered participant-level data; black dots with error bars indicate means with 95% confidence intervals.

We based our first test of the Dunning–Kruger effect on the approach followed by the original authors (Kruger and Dunning 1999). Like them, we split our sample into quartiles based on participants’ objective IQ. The original authors then derived their conclusions from a set of *t*-tests that compared the self-estimated and measured performance within each group—they only reported this for lowest and highest quartile—and a plot. Recent studies have used a comparable but more comprehensive ANOVA approach (e.g., West and Eaton 2019). Following them, we conducted one 2 (within: type of measure; self-estimated IQ vs. actual IQ) × 4 (between: performance quartile) ANOVA per domain. The relevant measure × quartile interaction effect was significant for all four domains (all *p* < .001, all η^2^_g_ ≥ .15; for full ANOVA results, see Table 3). Table 4 holds statistics on pairwise comparisons at the quartile-level. As can also be seen in Figure 1, the data showed a pattern indicative of a Dunning–Kruger effect for the majority of domains. That is, people in the lowest quartile showed the largest overestimation effects, while people in higher and particularly the highest quartiles tended to underestimate themselves. Only numerical intelligence exhibited a different pattern: here, self-estimates by those in the lowest quartile did not differ significantly from measured intelligence; people in the other three quartiles showed considerable effects of underestimation.

#### 3.4.2. Heteroscedasticity

**Figure 2:**
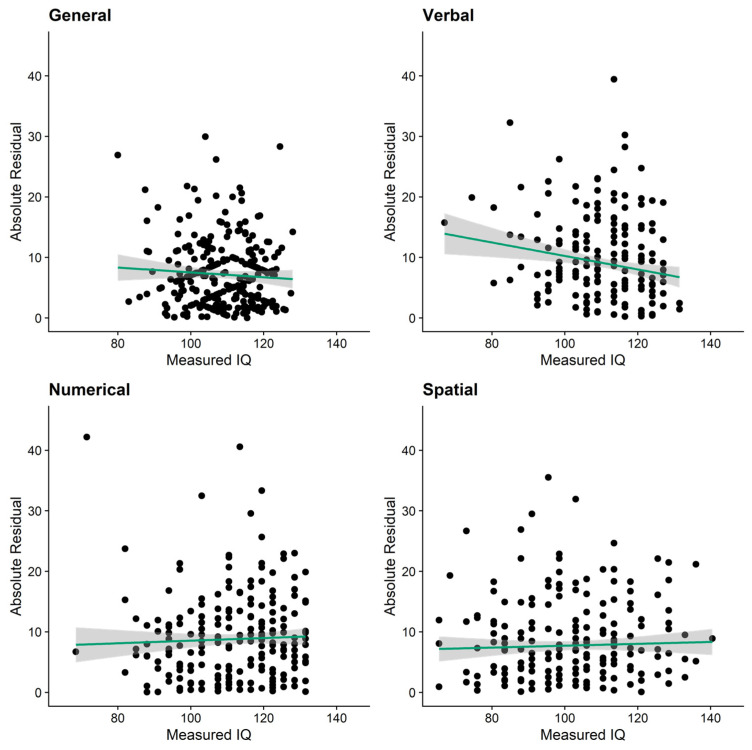
Dunning–Kruger effects: Glejser correlations of heteroscedasticity. Green lines represent linear associations with 95% confidence bands around them (shaded grey).

To further test for potential Dunning–Kruger effects, we used the recently proposed analyses methods proposed by Gignac and Zajenkowski (2020). The first analysis they suggested was the Glejser correlation (Glejser 1969)—an indicator of heteroscedasticity of residuals. To compute Glejser correlations, we predicted self-estimated from objectively measured IQs in linear regressions, converted the resulting residuals into absolute values, and ran correlations between these absolute residuals and objective IQs. According to Gignac and Zajenkowski (2020), a significantly negative Glejser correlation would indicate a Dunning–Kruger effect, as this would mean that smaller values in objectively measured intelligence are associated with larger absolute residuals. As depicted in Figure 2, we observed such a significant negative correlation for verbal intelligence (*r* = −.17, 95% BCa CI [−.29; −.05], *p* = .003) but none of the other domains (general intelligence: *r* = −.06, 95% BCa CI [−.22; .06], *p* = .308; numerical intelligence: *r* = .04, 95% BCa CI [−.16; .16], *p* = .668; spatial intelligence: *r* = .03, 95% BCa CI [−.08; .15], *p* = .551).

#### 3.4.3. Nonlinear Regression

**Figure 3:**
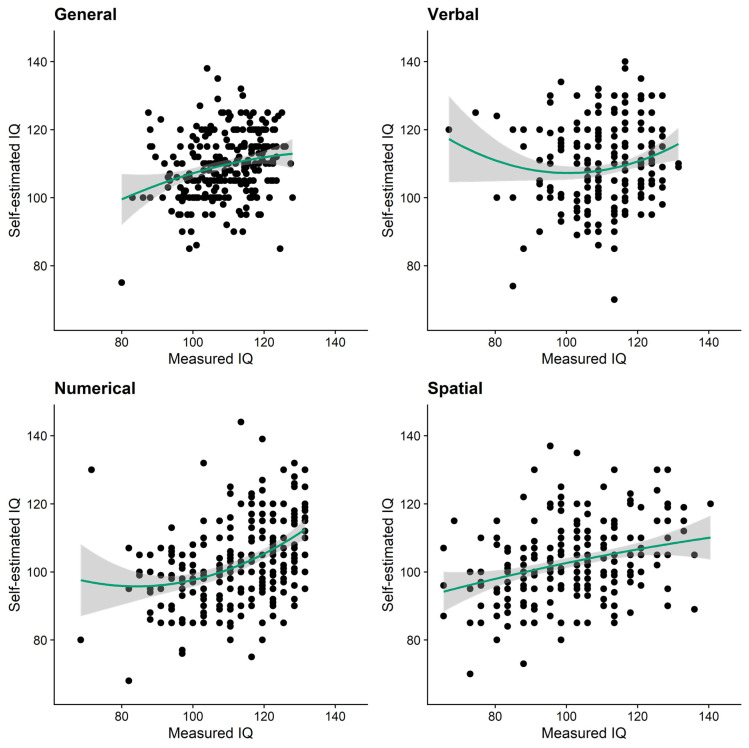
Dunning–Kruger effects: quadratic associations between measured and self-estimated intelligence. Note: green lines represent quadratic lines of best fit with 95% confidence bands around them (shaded grey).

The second method that Gignac and Zajenkowski (2020) proposed as an adequate test of the Dunning–Kruger effect is nonlinear regression. Thus, for each domain, we conducted hierarchical regression analyses with self-estimated IQ being predicted by the linear term of the objectively measured IQ in the first step and the quadratic term of the objectively measured IQ being added in the second step. Here, a Dunning–Kruger effect would be supported by a significant *R*² increase between steps and a significantly positive quadratic effect. Note that in this type of analysis, β-weights are not straightforwardly interpretable and semi-partial correlations should be considered instead (Gignac 2019). As visualized in Figure 3, for verbal and numerical intelligence there was some support for positive quadratic effects of IQ on self-estimates. Table 5 shows that, for both of these domains, the inclusion of the quadratic term led to significant increases in explained variance. The squared semi-partial correlations associated with the quadratic terms were also positive, indicating that the association between measured and self-estimated intelligence is larger at higher levels of measured intelligence. However, in both cases the bootstrapped confidence intervals around the regression weights crossed zero, questioning the robustness of these effects. For numerical intelligence, this quadratic effect was potentially driven by a single influential case (see Figure 3). After excluding this participant, neither the *R*² change nor the semi-partial correlation of the quadratic term were significant (for full results see Appendix A).

### 3.5. Exploratory Research Question

In our final research question, we wanted to explore whether people rather think of their strengths or their weaknesses when estimating their overall IQ. To test this, we correlated self-estimates of general intelligence with (1) the measured IQ in the domain in which participants had their personal best score, and (2) the measured IQ in the domain in which participants had their personal lowest score. In the majority of cases, the difference between participants’ best and worst domain was considerable: 74.38% showed a difference of more than 15 IQ-points. Across the whole sample, participants also seemed to rely more strongly on their best domain (*r* = .29, 95% BCa CI [.18; .42], *p* < .001) than on their weakest one (*r* = .12, 95% BCa CI [.00; .26], *p* = .053) when self-estimating their general intelligence. This difference was statistically significant (Williams’ *t*(278) = −3.05, *p* = .002). Arguably, small differences in people’s IQs between domains might not necessarily reflect strengths or weaknesses but could be due to measurement error. For this reason, we repeated the analysis for a sub-sample (*n* = 131) with an IQ difference between their best and weakest domain above the sample mean (*M* = 21.40). Here, the differences of correlations between the best (*r* = .23, 95% BCa CI [.05; .37], *p* = .009) and weakest (*r* = .15, 95% BCa CI [−.01; .32], *p* = .062) domains was smaller and no longer significant (Williams’ *t*(128) = −1.21, *p* = .230).

## 4. Discussion

In the present study, we aimed to investigate the accuracy of self-estimates of general, verbal, numerical, and spatial intelligence from various angles, but with a particular focus on potential Dunning–Kruger effects. In line with our preregistered expectations, self-estimates of intelligence showed mostly moderate correlational accuracy that was slightly higher in the numerical domain and lower in the verbal domain (see also Freund and Kasten 2012; Neubauer et al. 2018; Neubauer and Hofer 2021). This correlational pattern was virtually the same across three different operationalizations of self-estimates (a multi-item Likert-like scale covering multiple aspects of the respective intelligence facet, global Likert-like items from this scale, and single IQ-estimates) and two types of analyses (Pearson and intraclass correlations). As predicted, participants also rated their general intelligence as well as their intelligence on the three sub-facets to be above average (see also Heck et al. 2018; Visser et al. 2008). Somewhat unexpectedly, these high self-estimates did not constitute an overestimation: across the sample, participants underestimated their numerical intelligence and showed no significant over- or underestimation of their general, verbal, and spatial intelligence. Importantly, participants had to self-estimate their intelligence quotients with reference to the general population. As they were mostly highly educated, it stands to reason that their tendency to rate their intelligence as above average was in many cases not an overestimation but a rather accurate assessment (Heck et al. 2018; Visser et al. 2008). This corresponds to another study in which college students’ self-estimated performance on a variety of cognitive tests was rather close to their actual performance or constituted a slight underestimation (Ackerman and Wolman 2007).

Our participants’ knowledge about their own intelligence depended on their standing on the underlying ability—at least when operationalized as intelligence quartile: when it came to assessing their general, verbal, and spatial intelligence, those in the lowest respective intelligence quartile overestimated themselves the most, while particularly those in the highest quartile underestimated themselves. These findings are indicative of Dunning–Kruger effects (Kruger and Dunning 1999) and in line with studies across many ability domains (e.g., Kruger and Dunning 1999; von Stumm 2014; West and Eaton 2019). In our study, only numerical intelligence exhibited a different pattern, with rather accurate estimates in the lowest quartile and underestimation by the remaining groups. Based on participant feedback, we suspect that this could be due to the fact that, when self-estimating their numerical intelligence, many were considering more complex mathematical problems than the number series we applied as accuracy criterion. Of note, as people’s self-estimates showed only small-to-moderate correlations to objective intelligence criteria, there are likely regression-to-the-mean effects in these data. Thus, together with the above-average effects across aspects of intelligence, it is plausible that the Dunning–Kruger effects we found using this quartile-based approach are—at least partly—due to statistical artefacts (see Ackerman et al. 2002; Gignac and Zajenkowski 2020; Krueger and Mueller 2002).

Results on the Dunning–Kruger effect changed considerably when we used statistical methods that do not rely on artificial categorization of continuous data (i.e., nonlinear regression and a measure of heteroscedasticity; see also (Gignac and Zajenkowski 2020). For general and spatial intelligence, we neither found support for nonlinear associations between measured and self-estimated abilities nor for higher absolute residuals in low-performers’ estimates. While there was some indication for nonlinear associations between measured and self-estimated numerical intelligence, this effect was likely driven by a single influential case. The domain for which we found the most consistent—but still mixed—support for Dunning–Kruger effects was verbal intelligence: here, people at the lower end of the intelligence spectrum showed higher misestimation (i.e., absolute residuals) than those at the higher end. With *r* = −.17, this effect could be considered small to medium in the context of individual difference research (Gignac and Szodorai 2016; Gignac and Zajenkowski 2020). Moreover, there was some indication of quadratic effects between measured and self-estimated verbal intelligence. However, this finding also did not prove robust in bootstrapped analyses. Gignac and Zajenkowski (2020) recommended that authors should only consider data exhibiting both significant heteroscedasticity and a significant quadratic effect to be supportive of a Dunning–Kruger effect. Thus, future studies are needed to confirm or dispel this first, very tentative support for a Dunning–Kruger effect in verbal intelligence. Taken together, our results are well in line with past work that reported Dunning–Kruger effects for general intelligence using the quartile-based approach (Gignac and Zajenkowski 2020; von Stumm 2014) but only mixed evidence when using statistical approaches that do not require artificial categorization (Gignac and Zajenkowski 2020).

### 4.1. Implications

The present study adds to a growing literature questioning the robustness of the Dunning–Kruger effect. Recently, Gignac (2022) reported on the Dunning–Kruger effect in financial literacy. Just like in the present study, the effect was supported in quartile-based analyses but not in tests for nonlinearity or heteroscedasticity. That Dunning–Kruger effects are consistently detected in one type of analysis, but fail to emerge in other—likely more adequate—tests, conforms with accounts attributing the effect at least partly to statistical artefacts (e.g., Ackerman et al. 2002; Feld et al. 2017; Krajč and Ortmann 2008; Krueger and Mueller 2002; Nuhfer et al. 2016). Due to the large size of the Dunning–Kruger effect reported in some studies, Gignac and Zajenkowski (2020) concluded that it is likely not completely attributable to statistical artefacts but rather overestimated due to them. Of note, the authors of a recent study applied yet another type of statistical analyses—fitting Bayesian and performance-dependent models to their data—and did find support that low performers in the tasks originally applied by Kruger and Dunning (1999) were indeed worse judges of their own performance (Jansen et al. 2021). Nevertheless, the authors cautioned against generalizing from their results to potential Dunning–Kruger effects in other domains. It, thus, remains open whether such performance-dependent models would also show a good fit for intelligence test data such as those in our study.

Overall, it appears increasingly plausible that the Dunning–Kruger effect might be less ubiquitous than earlier work suggested. This raises questions about potential boundary conditions of the effect: what factors—apart from the analyses used to test for it—determine whether people show a Dunning–Kruger effect? Dunning (2011) already proposed that people’s oversight of their own incompetence depends on the type of skill that they have to assess. Similarly, Gignac and Zajenkowski (2020) acknowledged that Dunning–Kruger effects might emerge in some domains but not in others. In line with this, our data provide the first, tentative, supporting evidence that different aspects of intelligence might differ in how susceptible they are to Dunning–Kruger effects: it appears that (only) people with low verbal intelligence have particular difficulties in recognizing their shortcomings. While future work is still needed to confirm this effect, we already find it informative to speculate about what might make verbal intelligence different from the other intelligence domains. One often-discussed moderator of self-knowledge is the social desirability of the domain in question (e.g., John and Robins 1993; Vazire 2010): people likely have a harder time assessing themselves—and particularly their shortcomings—in very socially desirable domains, as these are thought to be more strongly related to self-esteem. Do people find high verbal intelligence more desirable than high numerical, spatial, or general intelligence? The comparatively low accuracy correlation for verbal intelligence would be in line with this assumption. In a current study, people indeed rated being verbally intelligent as more important to their sense of self-worth than being numerically or spatially intelligent (there was no comparable measure for general intelligence; (Hofer et al. 2021). It would, thus, be interesting to see how far social desirability—perhaps in addition to other discussed moderators such as task difficulty (Burson et al. 2006)—affects which domains are prone to elicit Dunning–Kruger effects.

Taken together, we believe that there are still many questions to be answered about people’s self-knowledge regarding their intelligence and other abilities. As people’s self-views are related to psychological adjustment (even though the literature is still not completely clear on the exact nature of this association; (Dufner et al. 2018; He and Côté 2019; Humberg et al. 2019; Kim et al. 2010; Kim and Chiu 2011) and likely guide important career and other life decisions (see Ackerman and Wolman 2007; Freund and Kasten 2012), we believe that it will remain important to conduct research on what people know about their own cognitive abilities. Our findings underline that this research will benefit from considering different operationalizations of accuracy and different aspects of intelligence instead of *g* alone, as these might yield rather different results. In our exploratory analyses, people’s self-estimates of their general intelligence correlated more highly with their IQ in their personal best domain than with their weakest one. Thus, people potentially differ in the intelligence facets on which they base their overall intellectual self-assessment, depending on their individual strengths and weaknesses—another reason for researchers and practitioners not to focus on self-estimates of *g* alone. Further interesting insights might be gained from explicitly asking participants how they derived their self-assessment. In view of the Dunning–Kruger effect’s popularity and the mixed results on its robustness, research on it continues to be important. Here, we concur with other authors (Gignac and Zajenkowski 2020; Jansen et al. 2021) that future work should refrain from splitting data into quartiles, as this procedure does not offer the kind of resolution needed to provide sufficient answers regarding this effect. There are likely more insights to be gained from using more adequate and easily implemented statistical methods described by Gignac and Zajenkowski (2020) or the modeling approach applied by Jansen et al. (2021).

### 4.2. Strengths and Limitations

We conducted an in-depth investigation into the accuracy of self-estimates of intelligence. To our knowledge, we were the first to test for the Dunning–Kruger effect with different statistical methods not only for general cognitive ability but for three central sub-facets of intelligence. While we consider our pre-registered methodology involving different domains, self-estimate measures, operationalizations of accuracy, and statistical approaches to be a particular strength, our study also comes with some limitations.

First, due to the COVID-19 crisis, it was not possible to conduct this study in the lab under normal supervised conditions. Instead, participants completed all measures online, which might have introduced error variance, particularly in the intelligence measures. We could not rule out cheating aside from excluding participants that admitted to doing so. However, it should be noted that participants had nothing to gain from cheating and were explicitly told that cheating would render their feedback worthless. The online testing might have also allowed for distractions, thus lowering performance. Nevertheless, we want to emphasize that most of our results are well in-line with those of comparable in-person studies. Second, on average, our sample scored quite highly on the majority of intelligence measures. This might be due to the rather old norms of our intelligence measure (Fay et al. 2001) not being adequate anymore because of the Flynn effect. If that was the case, the test overestimated people’s true intelligence (e.g., Trahan et al. 2014; but see Pietschnig and Voracek 2015 for recent declines in the Flynn effect). At the same time, it is quite likely that our highly educated convenience sample was indeed above average in their intelligence. Particularly for the investigation of Dunning–Kruger effects, a sample including a higher number of low performers would have been beneficial, since this group is at the very core of the proposed effect. However, we want to note that Gignac and Zajenkowski (2020) found comparable results for general intelligence in a more intellectually diverse sample. Third, our choice of intelligence measure could be questioned: while the ISA (Fay et al. 2001) is an often-applied, well-conceived, and standardized test, it does not differentiate well at the more extreme ends of the intelligence distribution. This does not appear to be a problem in our study—only very few participants scored at the lower or upper bounds—but future work involving a more diverse sample might want to consider other instruments. Finally, we based the timing of collecting self-estimates in our study (before the intelligence test) on Gignac and Zajenkowski (2020; but see also West and Eaton 2019), thereby deviating from earlier work on the Dunning–Kruger effect presenting self-estimates after performance tests (Kruger and Dunning 1999). Notably, studies using the same order we did also reported Dunning–Kruger effects—at least when applying classical quartile-based analyses (Gignac and Zajenkowski 2020; West and Eaton 2019). Moreover, meta-analytic evidence suggests that the timing of self-estimates has little effect on their accuracy (Freund and Kasten 2012; Zell and Krizan 2014). Overall, future replications involving in-person testing, a more nuanced intelligence measure, a sample including more low performers, and potentially presenting self-estimates after intelligence measures will determine how robust our results are.

### 4.3. Conclusions

Coming back to our initially posed question about how much people know about their own intelligence, the response that our results and past work suggest is “It depends”. When looking at correlational accuracy, people appear to be worst at judging their verbal intelligence and best at judging their numerical intelligence. However, even for self-estimated numerical intelligence the correlation with test performance was only at about .4, which is in line with the substantial body of evidence showing that one’s self-estimated ability level does not necessarily correspond very well to one’s objectively measured ability (e.g., Freund and Kasten 2012; Zell and Krizan 2014). This leads us—and other researchers (Ackerman and Wolman 2007; Freund and Kasten 2012)—to caution against using self-estimated intelligence as a stand-in for actual intelligence, be it in research or in applied settings such as career counselling. Our data also highlight the importance of looking at the accuracy of self-perceptions from different viewpoints: had we just looked at the mostly negligible mean differences between self-estimated and measured intelligence, we would have probably concluded that people, on average, have a rather accurate idea of their own cognitive abilities—perhaps apart from underestimating their numerical intelligence. Most importantly, we only found mixed evidence for Dunning–Kruger effects, particularly when we applied statistical methods that do not rely on assigning participants to performance quartiles. While there is an immense amount of literature speaking for Dunning–Kruger effects in many domains, our results and those of related work raise questions about the effect’s supposedly ubiquitous nature. Instead, our findings might indicate that some performance domains—in our case, verbal intelligence—are more susceptible to Dunning–Kruger effects than others. Future studies with samples including a larger number of low performers are needed to confirm this and could further provide insights into potential reasons for these differences between domains. Considering the high popularity of the Dunning–Kruger effect in research and pop-culture alike, as well as its potential real-life consequences, research in this area will continue to be important.

## Figures and Tables

**Table 1:** Descriptive statistics and intercorrelations of measured IQs, single-item self-estimated IQs, and multi-item intelligence self-estimates.

Variable		Min-Max	*M* (*SD*)	1	2	3	4	5	6	7	8	9	10
1.	General IQ	80.00–128.00	108.78 (9.06)										
2.	Verbal IQ	67.00–131.50	110.96 (10.27)	.57									
3.	Numerical IQ	68.50–131.50	113.28 (13.10)	.77	.22								
4.	Spatial IQ	65.50–140.50	102.11 (14.46)	.78	.16	.38							
5.	SE General IQ	75.00–138.00	109.29 (9.40)	.25	.18	.24	.11						
6.	SE Verbal IQ	70.00–140.00	109.15 (11.28)	.09	.10	.12	−.02	.64					
7.	SE Numerical IQ	68.00–144.00	103.35 (12.24)	.40	.19	.40	.26	.63	.18				
8.	SE Spatial IQ	70.00–137.00	102.90 (10.58)	.32	.20	.18	.29	.55	.17	.54			
9.	SE Verbal Multi-Item	1.70–4.90	3.49 (.61)	.14	.18	.15	−.01	.40	.65	.11	.08		
10.	SE Numerical Multi-Item	1.00–5.00	3.03 (.98)	.40	.16	.40	.28	.34	−.09	.76	.39	.12	
11.	SE Spatial Multi-Item	1.22–5.00	3.16 (.80)	.15	.11	.01	.20	.19	−.07	.21	.66	.14	.38

Note: SE = Self-estimated. With *n* = 281, all *r* ≥ .12 are significant at *p* < .05 and all *r* ≥ .19 are significant at *p* < .001.

**Table 2:** Correlational accuracy of different self-estimate measures.

Domain	SE (IQ)	SE (Multi-Item)	SE (Last Item)
General	.25		
	[.12, .38]		
	*p* < .001		
Verbal	.10	.19	.17
	[−.02, .23]	[.08, .28]	[.05, .28]
	*p* = .100	*p* < .001	*p* = .001
Numerical	.40	.40	.34
	[.27, .49]	[.28, .49]	[.21, .44]
	*p* = .003	*p* = .001	*p* = .002
Spatial	.29	.20	.30
	[.18, .40]	[.08, .32]	[.18, .40]
	*p* = .001	*p* = .001	*p* = .001

Note: *n* = 281. SE (IQ) = self-estimated IQ (Bonferroni-correction .05/4: *p <* .013). SE (Multi-Item) = mean of item responses to the multi-item self-estimate scale (Bonferroni-correction .05/3: *p <* .017). SE (Last Item) = response to last, global item of the multi-item self-estimate scale (Bonferroni-correction .05/3: *p <* .017). Values in brackets are 95% BCa confidence intervals based on 2000 bootstrap samples.

**Table 3:** Dunning–Kruger effects: main and interaction effects of 2 (measure: self-estimated vs. measured IQ) × 4 (IQ quartile) analyses of variance.

Domain	Effect	*F*	*df*_1_	*df*_2_	*p*	η^2^_g_
General	Quartile	116.69	3	277	<.001	.391
	Measure	0.78	1	277	.378	.001
	Quartile × Measure	37.86	3	277	<.001	.168
Verbal	Quartile	84.46	3	277	<.001	.296
	Measure	5.78	1	277	.017	.011
	Quartile × Measure	30.21	3	277	<.001	.150
Numerical	Quartile	174.02	3	277	<.001	.501
	Measure	200.55	1	277	<.001	.253
	Quartile × Measure	38.72	3	277	<.001	.164
Spatial	Quartile	178.22	3	277	<.001	.516
	Measure	1.54	1	277	.216	.002
	Quartile × Measure	96.01	3	277	<.001	.318

Note: *n* = 281.

**Table 4:** Dunning–Kruger effects: pairwise comparisons of self-estimated vs. measured IQ per IQ quartile.

Domain	Quartile	*t*	*df*	*M*_diff_	95% BCa CI	*p*	*d*
General	80–103	6.78	72	8.32	[5.95; 10.68]	<.001 *	0.79
	103.5–109	2.93	68	3.33	[1.20; 5.65]	<.001 *	0.35
	109.5–116	−2.01	73	−2.20	[−4.39; 0.03]	.055	−0.23
	116.5–128	−7.46	64	−8.18	[−10.38; −6.12]	<.001 *	−0.92
Verbal	67–106	4.76	96	7.20	[4.36; 10.09]	<.001 *	0.48
	106.5–113.5	−2.45	74	−3.44	[−6.09; −0.86]	.012 *	−0.28
	114–116.5	−2.68	42	−4.64	[−7.92; −1.08]	.018	−0.41
	117–131.5	−9.22	65	−11.36	[−13.71; −8.86]	<.001 *	−1.13
Numerical	68.5–103	0.74	77	1.05	[−1.58; 3.96]	.442	0.08
	103.5–116.5	−6.90	76	−9.31	[−11.97; −6.64]	<.001 *	−0.79
	117–122.5	−10.13	58	−16.04	[−19.13; −12.91]	<.001 *	−1.32
	123–131.5	−14.41	66	−18.02	[−20.26; −15.60]	<.001 *	−1.76
Spatial	65.5–91	11.26	79	13.98	[11.67; 16.36]	<.001 *	1.26
	91.5–103	3.91	75	5.03	[2.54; 7.56]	<.001 *	0.45
	103.5–113.5	−6.12	77	−6.69	[−8.90; −4.54]	<.001 *	−0.69
	114–140.5	−10.15	46	−16.09	[−19.15; −12.95]	<.001 *	−1.48

Note: *n* = 281. * = significant after Bonferroni-correction (.05/4: *p* < .013). Values for quartile show the upper and lower bound of each quartile in IQ-points. Confidence intervals are based on 2000 bootstrap samples. Positive values for mean differences, *t*, and *d* indicate that self-estimated IQ is higher than measured IQ (i.e., overestimation).

**Table 5:** Hierarchical regressions with linear and quadratic effects of measured intelligence in different domains on respective self-estimates of intelligence.

Domain	Predictor	*b*	95% CI*_b_*	β	95% CI_β_	*sr*²	95% CI*_sr_*_²_	*r*	*R*² [95% CI]	Δ*R*² [95% CI]
General	Step 1									
	(Intercept)	81.42 **	[66.74, 95.86]						.061 **	
	IQ	0.26 **	[0.13, 0.39]	.25	[.12, .37]	.06	[.02, .13]	.25 **	[.02, .13]	
	Step 2									
	(Intercept)	39.01	[−108.62, 220.43]						.063 **	.002
	IQ	1.05	[−2.25, 3.85]	1.02	[−2.21, 3.63]	.00	[.00, .04]	.25 **	[.02, .16]	[.00, .04]
	IQ²	−0.00	[−0.02, 0.01]	−.77	[−3.38, 2.42]	.00	[.00, .04]	.24 **		
Verbal	Step 1									
	(Intercept)	96.81 **	[79.90, 112.15]						.010	
	IQ	0.11	[−0.02, 0.26]	.10	[−.02, .23]	.01	[.00, .05]	.10	[.00, .05]	
	Step 2									
	(Intercept)	197.07 **	[68.37, 281.14]						.028 *	.018 *
	IQ	−1.79 *	[−3.31, 0.54]	−1.63	[−3.00, .46]	.02	[.00, .05]	.10	[.01, .07]	[.00, .06]
	IQ²	0.01 *	[−0.00, 0.02]	1.73	[−.28, 3.12]	.02	[.00, .06]	.11		
Numerical	Step 1									
	(Intercept)	61.24 **	[48.65, 74.45]						.158 **	
	IQ	0.37 **	[0.25, 0.48]	.40	[.28, .50]	.16	[.08, .25]	.40 **	[.08, .25]	
	Step 2									
	(Intercept)	148.79 **	[42.72, 268.27]						.173 **	.015 *
	IQ	−1.26	[−3.43, 0.66]	−1.35	[−3.70, .69]	.01	[.00, .07]	.40 **	[.11, .27]	[.00, .08]
	IQ²	0.01 *	[−0.00, 0.02]	1.75	[−.25, 4.06]	.02	[.00, .08]	.41 **		
Spatial	Step 1									
	(Intercept)	81.06 **	[72.00, 90.31]						.085 **	
	IQ	0.21 **	[0.12, 0.30]	.29	[.17, .40]	.09	[.03, .16]	.29 **	[.03, .16]	
	Step 2									
	(Intercept)	72.94 **	[18.86, 121.24]						.086 **	.000
	IQ	0.38	[−0.58, 1.44]	.51	[−.82, 1.96]	.00	[.00, .03]	.29 **	[.03, .17]	[.00, .02]
	IQ²	−0.00	[−0.01, 0.00]	−.22	[−1.67, 1.12]	.00	[.00, .02]	.29 **		

Note: *n* = 281. IQ = Intelligence Quotient. Values in brackets represent 95% percentile bootstrap confidence intervals based on 2000 samples. Significant *b*s also indicate significant βs and *sr*²s. * indicates *p* < .05. ** indicates *p* < .01.

## Data Availability

The data presented in this study are openly available on the Open Science Framework at https://doi.org/10.17605/OSF.IO/MJD8E.

## Note

1 Unfortunately, we had overlooked this discrepancy at the planning stage. However, we believe that the self-estimates of the remaining participants are still valid as they were either within the bounds of the intelligence tests or would have also corresponded to an over-/underestimation with intelligence tests with a broader range (e.g., a self-estimated IQ of 138 compared to a measured one of 104).
